# Implementation of novel antithrombotic treatment strategies and outcome improvements in patients with myocardial infarction and atrial fibrillation over 20 years: a nationwide cohort study

**DOI:** 10.1093/ehjcvp/pvag027

**Published:** 2026-04-27

**Authors:** Christian Losciale, Lars Lindhagen, Lars Wallentin, Tomas Jernberg, Joakim Alfredsson, Jonas Oldgren, Gorav Batra

**Affiliations:** Department of Medicine, Nyköpings Lasarett, 611 39 Nyköping, Sweden; Department of Medical Sciences, Cardiology, Uppsala University, 751 85 Uppsala, Sweden; Uppsala Clinical Research Center, Uppsala University, 751 85 Uppsala, Sweden; Department of Medical Sciences, Cardiology, Uppsala University, 751 85 Uppsala, Sweden; Uppsala Clinical Research Center, Uppsala University, 751 85 Uppsala, Sweden; Department of Clinical Sciences, Danderyd Hospital, Karolinska Institute, 182 88 Stockholm, Sweden; Department of Health, Medicine and Caring Sciences, Linköping University, 581 83 Linköping, Sweden; Department of Cardiology, Linköping University, 581 83 Linköping, Sweden; Department of Medical Sciences, Cardiology, Uppsala University, 751 85 Uppsala, Sweden; Uppsala Clinical Research Center, Uppsala University, 751 85 Uppsala, Sweden; Department of Medical Sciences, Cardiology, Uppsala University, 751 85 Uppsala, Sweden; Uppsala Clinical Research Center, Uppsala University, 751 85 Uppsala, Sweden

**Keywords:** Myocardial infarction, Atrial fibrillation, Oral anticoagulants

## Abstract

**Aims:**

Patients with acute myocardial infarction (MI) and atrial fibrillation (AF) present challenges in antithrombotic treatment. Effects of newer treatment strategies on outcomes remain uncertain. We assessed implementation of antithrombotic treatment and occurrence of cardiovascular (CV) events and major bleeding in patients with MI and AF.

**Methods and results:**

Nationwide data from SWEDEHEART and national health registries were used to identify 71 513 survivors of an acute MI with AF between 2000 and 2021. Changes in antithrombotic therapies over time in 2-year cohorts were analysed, and associations with 1-year outcomes were assessed. The outcomes were ischaemic stroke or systemic embolism, MI, major bleeding, CV mortality and all-cause mortality. Logistic regression was used to standardize for changes in patient characteristics and treatment over time. Oral anticoagulant use increased from 21% in 2000–2001 to 75% in 2020–2021, largely due to adoption of direct oral anticoagulants (DOACs) after the first DOAC approval in 2011, most often combined with a single antiplatelet. Over time, particularly in the last decade, 1-year risk of ischaemic stroke or systemic embolism declined from 6.0% to 2.1%, and CV mortality decreased from 19.8% to 10.5%. Major bleeding increased from 3.9% to 7.6% in 2014–2015, then declined to 6.4% in 2020–2021, simultaneously with adoption of single rather than dual antiplatelet therapy in combination with DOAC.

**Conclusion:**

Between 2000 and 2021, among patients with MI and AF, we observed reductions in 1-year risks of ischaemic stroke or systemic embolism and CV mortality, occurring during a period of increased prescription of oral anticoagulants, particularly DOACs.

**Key question and findings:**

Key Question

Have recent clinical trial findings on antithrombotic therapy in patients with myocardial infarction and concomitant atrial fibrillation been implemented, and have they resulted in any improvement in 1-year outcomes?

Key Finding

In patients with myocardial infarction and atrial fibrillation, we observed substantial reductions in 1-year risks of ischaemic stroke or systemic embolism and cardiovascular mortality, associated with increased prescribing of oral anticoagulants, particularly DOACs.

## Graphical Abstract

**Graphical Abstract:**
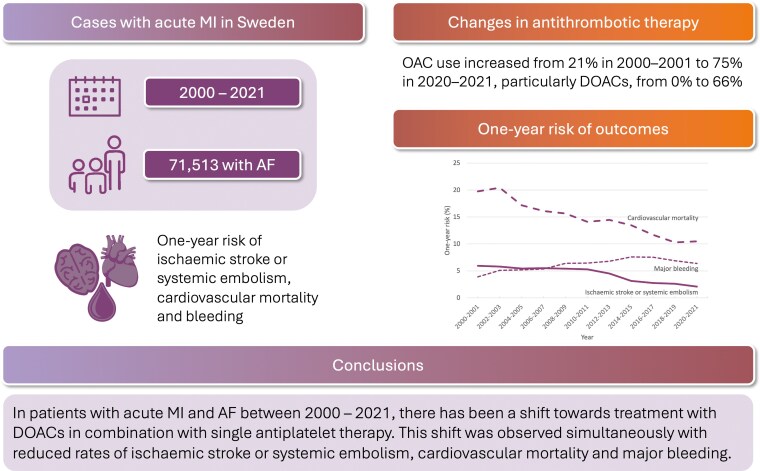

## Introduction

Atrial fibrillation (AF) is the most common sustained arrhythmia and is associated with an increased risk of ischaemic stroke and systemic embolism, heart failure, and death.^1,2^ Oral anticoagulation with vitamin K antagonists (VKAs) such as warfarin have, since the 1990s, been used for prevention of ischaemic stroke and systemic embolism,^3^ but is associated with an increased risk of bleeding, including intracranial haemorrhage, and require careful monitoring due to their narrow therapeutic window.^4,5^ Over the past 15 years, direct oral anticoagulants (DOACs) have emerged as an at least equally effective but safer alternatives to VKAs in patients with AF, particularly due to stable anticoagulation and lower risk of intracranial bleeding.^6–9^

In patients with acute myocardial infarction (MI) undergoing percutaneous coronary intervention (PCI), dual antiplatelet therapy (DAPT) combining aspirin and a P2Y_12_ inhibitor, has been proven effective since 2001 in reducing risk of recurrent MI and cardiovascular (CV) death.^10^ Patients with both acute MI and AF present a therapeutic dilemma, as DAPT alone does not provide sufficient protection against ischaemic stroke and systemic embolism.^11^ The addition of an oral anticoagulant (OAC) to DAPT, referred to as triple therapy, effectively reduces the risk of ischaemic stroke but is associated with a significantly increased risk of bleeding, including life-threatening events such as intracranial haemorrhage.^12–14^ To address this issue, several randomized controlled trials (RCTs) have primarily evaluated the safety and, to some extent, the efficacy of dual antithrombotic therapy, in which either a VKA or a DOAC was combined with a P2Y_12_ inhibitor and/or aspirin. These studies demonstrated that dual therapy was associated with a lower risk of severe bleeding compared to triple therapy but were not sufficiently powered to conclusively assess the efficacy of dual antithrombotic therapy on CV and thromboembolic outcomes.^15–18^ Still, since early 2020s, clinical practice guidelines from the European Society of Cardiology, the American College of Cardiology, and the American Heart Association recommend an early transition from triple therapy to dual therapy with a DOAC and a single antiplatelet agent in most patients after acute MI with concomitant AF.^19,20^

In this observational study, based on the Swedish registry of acute MI over the past two decades, we aimed to evaluate nationwide temporal trends in antithrombotic treatment strategies and associated 1-year clinical outcomes among patients with acute MI and AF.

## Methods

### Study population and data sources

The Swedish Web-System for Enhancement and Development of Evidence-Based Care in Heart Disease Evaluated According to Recommended Therapies (SWEDEHEART) register is a nationwide quality registry for CV care, including MI and coronary interventions. It has been validated in previous studies.^21^ Data for this study were obtained from SWEDEHEART and enriched with information on concomitant diseases upon hospital admission, including a history of AF, from the Swedish National Patient Register, using the International Classification of Diseases (ICD) system (see Supplementary material online, *Table S1*).^22^ Outcome data were retrieved from the Swedish National Patient Register and the National Cause of Death Register (see Supplementary material online, *Table S2*).

All patients registered with an acute MI in SWEDEHEART were included. Exclusion criteria comprised age <18 years, unknown admission or discharge date, and in-hospital death. Patients were included in 2-year blocks between 2000 and 2021, and for patients with more than one hospital admission for acute MI within the same 2-year block, only the first admission was considered. The study cohort included patients with acute MI and either AF before or in association with the hospitalization for MI. A comparison cohort for sensitivity analysis included 1:1 matched patients with acute MI but without concomitant AF.

Data on antithrombotic treatment and other secondary preventive medications at discharge were extracted from SWEDEHEART. Antithrombotic medication was categorized into eight groups: (i) single antiplatelet therapy (SAPT) only, (ii) DAPT only, (iii) warfarin only, (iv) warfarin + SAPT, (v) warfarin + DAPT, (vi) DOAC only, (vii) DOAC + SAPT, or (viii) DOAC + DAPT.

### Data management and ethics

Data linkage was performed by the Swedish National Board of Health and Welfare, and pseudonymized data were provided to the researchers. All data management and statistical analyses were conducted by the researchers in collaboration with data managers and statisticians at Uppsala Clinical Research Center, Uppsala, Sweden, using R Statistics version 4.2.1. All patients in SWEDEHEART were informed about their participation in the national quality registry and had the option to opt out. The Swedish Ethical Review Authority approved the study (application number: 2020-04252).

### Outcomes

The outcomes of interest were the composite of ischaemic stroke or systemic embolism, CV mortality, all-cause mortality, acute MI, and major bleeding (intracranial, gastrointestinal, genitourinary, and other major bleeding requiring hospitalization) (see Supplementary material online, *Table S2*). All outcomes were assessed during a 1-year follow-up period after hospital discharge for index MI and were hence referred to as 1-year risks.

### Statistical analysis

Results were reported in accordance with the Strengthening the Reporting of Observational Studies in Epidemiology guidelines (see Supplementary material online, *Figure S1*). The population was divided into 2-year blocks, beginning with 2000–2001 and ending with 2020–2021, and individual data were analysed. Patient characteristics were summarized as medians with interquartile ranges for continuous variables and as percentages for categorical variables. Data on antithrombotic medication use over time were reported as numbers and percentages and illustrated using 100% stacked column charts. Changes in the 1-year risks over time were depicted using line graphs, while time-to-event data were presented with Kaplan–Meier curves. Standardization was conducted using logistic regression models to adjust for variations in patient demographics, comorbidities, and treatments over the observation period. This method allowed us to hypothetically transfer patients back to a previous admission year and adjustments were performed to account for temporal changes in patient characteristics and treatment strategies using a stepwise approach: (i) Demographics and baseline comorbidities, type of MI (STEMI, NSTEMI); (ii) pre- or in-hospital revascularization; (iii) warfarin at discharge; (iv) DOAC at discharge; and (v) other secondary preventive discharge medications, excluding oral anticoagulants (see Supplementary material online, *Table S3*). In a sensitivity analysis, we performed 1:1 propensity score matching using logistic regression to select a representative cohort of acute MI patients without concomitant AF but with similar baseline characteristics to the main AF population, in order to compare 1-year outcomes. Matching was based on the same demographic and baseline comorbidities as in the standardization models described above (see Supplementary material online, *Figure S2*). Missing data were minimal for most covariates (see Supplementary material online, *Table S4*). Multiple imputation, generating three imputed datasets using the chained equations method, was applied under the missing-at-random assumption.

## Results

### Baseline characteristics

**Figure 1:**
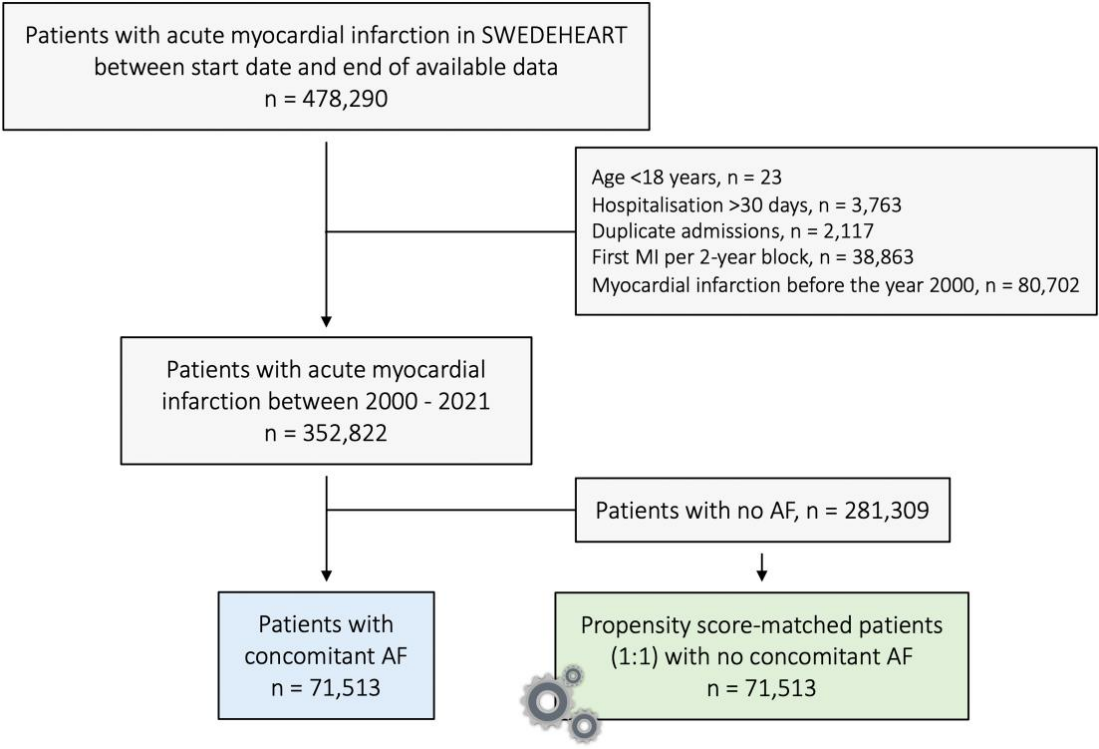
Consort diagram.

Between 1 January 2000 and 31 December 2021, a total of 352 822 cases with MI in Sweden were recorded in SWEDEHEART. Among them, 71 513 (20.3%) had either a history of AF or new-onset AF during the index MI event, forming the main study population, as shown in the flow chart (*Figure 1*). Baseline characteristics for patients with acute MI and AF, stratified by 2-year blocks, are summarized in *Table 1*. The median age of the study population with MI and concomitant AF was 79 years, with 60.9% being men. In total, 74.8% were diagnosed with non-ST-elevation MI (NSTEMI) and 25.2% with ST-elevation MI (STEMI). A history of AF prior to admission for the index MI was documented in 49.9% of the study population, while new-onset AF accounted for the remaining 50.1%. There were several changes in baseline characteristics and in-hospital treatment during the 22-year study period, including an increasing proportion of cases with obesity, hypertension, and diabetes, as well as a higher number undergoing coronary angiography, PCI, and CABG (*Table 1*).

**Table 1:** Baseline demographics and characteristics in 2-year blocks for patients with acute MI and concomitant AF

Characteristics	2000–2001	2002–2003	2004–2005	2006–2007	2008–2009	2010–2011	2012–2013	2014–2015	2016–2017	2018–2019	2020–2021
	(*n* = 5970)	(*n* = 6789)	(*n* = 6789)	(*n* = 7107)	(*n* = 6909)	(*n* = 6679)	(*n* = 6688)	(*n* = 6408)	(*n* = 6334)	(*n* = 6169)	(*n* = 5671)
Demographics											
Age, years, median (IQR)	78 (72–83)	79 (72–84)	79 (73–84)	79 (72–85)	80 (72–85)	79 (72–85)	80 (72–86)	79 (71–85)	78 (71–85)	78 (71–84)	78 (72–84)
Sex, male, *n* (%)	3638 (60.9%)	3962 (58.4%)	3963 (58.4%)	3963 (58.4%)	4071 (58.9%)	4071 (58.9%)	4071 (58.9%)	4071 (58.9%)	4010 (63.3%)	4039 (65.5%)	3687 (65.0%)
Obesity (BMI ≥30 kg/m^2^), *n* (%) [*n* = 49 806]	108 (16.0%)	282 (16.5%)	523 (16.5%)	735 (17.0%)	839 (18.0%)	1165 (20.1%)	1272 (20.7%)	1386 (23.0%)	1406 (23.3%)	1412 (24.1%)	1332 (24.6%)
Smoking, *n* (%) [*n* = 63 708]	718 (13.6%)	764 (12.6%)	768 (13.2%)	810 (13.0%)	771 (12.8%)	779 (13.3%)	738 (12.4%)	697 (12.1%)	743 (12.8%)	722 (12.7%)	669 (12.8%)
Medical history, *n* (%)											
Hypertension	2844 (47.6%)	3545 (52.2%)	3847 (56.7%)	4492 (63.2%)	4726 (68.4%)	4875 (73.0%)	5110 (76.4%)	5061 (79.0%)	5142 (81.2%)	5071 (82.2%)	4702 (82.9%)
Diabetes	1594 (26.7%)	1792 (26.4%)	1846 (27.2%)	2035 (28.6%)	1940 (28.1%)	1976 (29.6%)	2048 (30.6%)	2079 (32.4%)	2124 (33.5%)	2029 (32.9%)	1983 (35.0%)
Heart failure	2724 (45.6%)	3088 (45.5%)	3061 (45.1%)	3054 (43.0%)	2907 (42.1%)	2800 (41.9%)	2672 (40.0%)	2506 (39.1%)	2340 (36.9%)	2099 (34.0%)	1901 (33.5%)
COPD	470 (7.9%)	591 (8.7%)	629 (9.3%)	667 (9.4%)	666 (9.6%)	670 (10.0%)	709 (10.6%)	729 (11.4%)	730 (11.5%)	768 (12.4%)	666 (11.7%)
Prior MI	2313 (38.7%)	2738 (40.3%)	2727 (40.2%)	2878 (40.5%)	2746 (39.7%)	2708 (40.5%)	2693 (40.3%)	2537 (39.6%)	2476 (39.1%)	2326 (37.7%)	2081 (36.7%)
Prior stroke	1019 (17.1%)	1232 (18.1%)	1404 (20.7%)	1573 (22.1%)	1452 (21.0%)	1408 (21.1%)	1422 (21.3%)	1209 (18.9%)	1141 (18.0%)	1045 (16.9%)	945 (16.7%)
Prior systemic embolism	56 (0.9%)	81 (1.2%)	80 (1.2%)	80 (1.1%)	90 (1.3%)	72 (1.1%)	97 (1.5%)	75 (1.2%)	92 (1.5%)	78 (1.3%)	73 (1.3%)
Peripheral vascular disease	503 (8.4%)	607 (8.9%)	633 (9.3%)	654 (9.2%)	628 (9.1%)	638 (9.6%)	738 (11.0%)	684 (10.7%)	667 (10.5%)	610 (9.9%)	566 (10.0%)
Cancer (within 3 years)	276 (4.6%)	319 (4.7%)	358 (5.3%)	359 (5.1%)	373 (5.4%)	414 (6.2%)	479 (7.2%)	449 (7.0%)	419 (6.6%)	421 (6.8%)	333 (5.9%)
Prior major bleeding	376 (6.3%)	491 (7.2%)	587 (8.6%)	621 (8.7%)	635 (9.2%)	628 (9.4%)	669 (10.0%)	675 (10.5%)	665 (10.5%)	672 (10.9%)	621 (11.0%)
CHA_2_DS_2_-VASc score, *n* (%)											
1 point	174 (2.9%)	186 (2.7%)	167 (2.5%)	155 (2.2%)	172 (2.5%)	145 (2.2%)	132 (2.0%)	135 (2.1%)	130 (2.1%)	141 (2.3%)	146 (2.6%)
2 points	449 (7.5%)	455 (6.7%)	408 (6.0%)	456 (6.4%)	447 (6.5%)	423 (6.3%)	430 (6.4%)	384 (6.0%)	375 (5.9%)	392 (6.4%)	361 (6.4%)
3 points	958 (16.0%)	1023 (15.1%)	967 (14.2%)	990 (13.9%)	885 (12.8%)	853 (12.8%)	813 (12.2%)	845 (13.2%)	838 (13.2%)	839 (13.6%)	693 (12.2%)
≥ 4 points	4389 (73.5%)	5125 (75.5%)	5247 (77.3%)	5506 (77.5%)	5405 (78.2%)	5258 (78.7%)	5313 (79.4%)	5044 (78.7%)	4991 (78.8%)	4797 (77.8%)	4471 (78.8%)
In-hospital course, *n* (%)											
Type of MI [*n* = 70 853]											
NSTEMI	4129 (72.0%)	4932 (75.9%)	5183 (77.2%)	5546 (78.4%)	5142 (74.7%)	5041 (75.5%)	5055 (75.6%)	4837 (75.5%)	4696 (74.1%)	4403 (71.4%)	4056 (71.5%)
STEMI	1608 (28.0%)	1562 (24.1%)	1530 (22.8%)	1532 (21.6%)	1740 (25.3%)	1638 (24.5%)	1633 (24.4%)	1571 (24.5%)	1638 (25.9%)	1766 (28.6%	1615 (28.5%)
New-onset AF	3898 (65.3%)	3923 (57.8%)	3681 (54.2%)	3580 (50.4%)	3323 (48.1%)	3129 (46.8%)	3047 (45.6%)	2869 (44.8%)	2921 (46.1%)	2817 (45.7%)	2671 (47.1%)
Thrombolysis	1011 (17.2%)	787 (12.1%)	405 (6.0%)	165 (2.3%)	105 (1.5%)	73 (1.1%)	50 (0.7%)	56 (0.9%)	38 (0.6%)	39 (0.6%)	37 (0.7%)
Coronary angiography	632 (10.6%)	1395 (20.5%)	2469 (36.4%)	3312 (46.6%)	3576 (51.8%)	3865 (57.9%)	4094 (61.2%)	4193 (65.4%)	4557 (71.9%)	4675 (75.8%)	4417 (77.9%)
PCI	486 (8.1%)	886 (13.1%)	1647 (24.3%)	2186 (30.8%)	2443 (35.4%)	2603 (39.0%)	2999 (44.8%)	3172 (49.5%)	3588 (56.6%)	3707 (60.1%)	3591 (63.3%)
CABG	78 (1.3%)	95 (1.4%)	121 (1.8%)	140 (2.0%)	151 (2.2%)	343 (5.1%)	219 (3.3%)	219 (3.4%)	216 (3.4%)	210 (3.4%)	195 (3.4%)
eGFR, median (IQR)	81 (81–81)	56 (42–72)	58 (43–75)	60 (43–78)	60 (43–78)	62 (45–79)	61 (44–79)	63 (45–81)	64 (46–81)	65 (47–82)	66 (47–82)
Medication at discharge, *n* (%)											
Warfarin	1217 (21.0%)	1331 (20.2%)	1332 (19.8%)	1491 (21.0%)	1626 (23.6%)	1780 (26.7%)	2282 (34.2%)	2425 (37.9%)	1726 (27.3%)	829 (13.5%)	478 (8.4%)
DOAC	—	—	—	—	—	1 (0.0%)	38 (0.6%)	712 (11.1%)	2147 (33.9%)	3332 (54.0%)	3743 (66.0%)
Aspirin [*n* = 71 094]	4337 (74.3%)	4965 (75.1%)	5321 (78.9%)	5753 (81.1%)	5668 (82.1%)	5645 (84.6%)	5341 (79.9%)	4472 (69.9%)	4248 (67.2%)	3738 (60.7%)	2651 (46.8%)
P2Y_12_ inhibitors [*n* = 71 095]	516 (8.8%)	1661 (25.1%)	2798 (41.5%)	3749 (52.8%)	4120 (59.7%)	4266 (63.9%)	4537 (67.9%)	4380 (68.4%)	4433 (70.1%)	4458 (72.4%)	4084 (72.1%)
Beta blockers [*n* = 71 088]	4574 (78.3%)	5428 (82.2%)	5739 (85.1%)	6171 (87.0%)	6097 (88.3%)	5999 (89.9%)	5883 (88.1%)	5682 (88.7%)	5625 (89.0%)	5330 (86.5%)	4817 (85.1%)
ACE inhibitor/ARB	3143 (52.6%)	3629 (53.5%)	4184 (61.6%)	4770 (67.1%)	4920 (71.2%)	4956 (74.2%)	4933 (73.8%)	4852 (75.7%)	4870 (76.9%)	4806 (77.9%)	4504 (79.4%)
Statins [*n* = 70 957]	1994 (34.5%)	3052 (46.6%)	3849 (57.2%)	4743 (66.9%)	5022 (72.8%)	5082 (76.2%)	5095 (76.2%)	4994 (78.0%)	5146 (81.4%)	5128 (83.3%)	4831 (85.3%)
Ezetimibe [*n* = 70 848]	0 (0.0%)	0 (0.0%)	25 (0.4%)	79 (1.1%)	76 (1.1%)	77 (1.2%)	89 (1.3%)	108 (1.7%)	200 (3.2%)	331 (5.4%)	532 (9.4%)

Values are in median [interquartile range (IQR)] for continuous variables and numbers (%) for categorical variables. Information of missing data is available in Supplementary material online, *Table S5*. Abbreviations: ACE, angiotensin-converting enzyme; AF, atrial fibrillation; ARB, angiotensin receptor blockers; BMI, body mass index; CHA2DS2-VASc, congestive heart failure, hypertension, age, diabetes, stroke, vascular disease, sex; CABG, coronary artery bypass graft surgery; COPD, chronic obstructive pulmonary disease; DOAC, direct oral anticoagulants; MI, myocardial infarction; NSTEMI, non-ST-segment elevation myocardial infarction; PCI, percutaneous coronary intervention; STEMI, ST-segment elevation myocardial infarction.

### Changes in antithrombotic treatment at discharge over time

**Figure 2:**
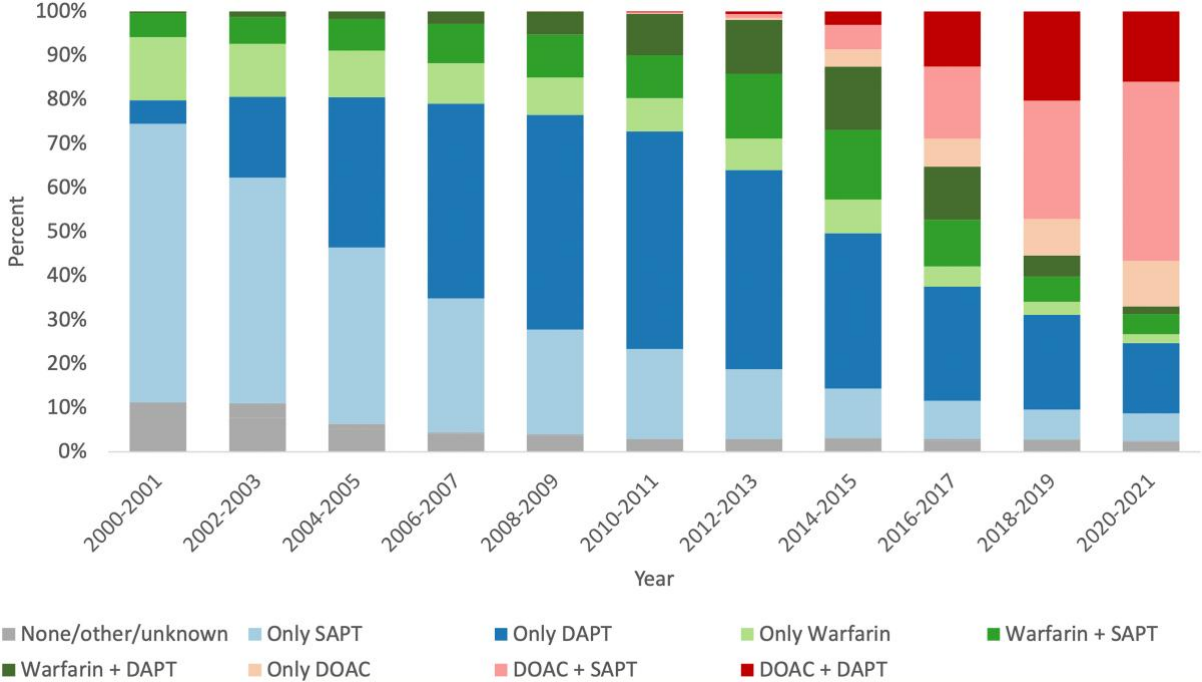
Stacked chart diagram of prescribed antithrombotic treatment at discharge among cases with acute MI and concomitant AF between 2000 and 2021.

Between 2000 and 2021, the overall prescription of OAC alone or in any combination with antiplatelets increased among patients with MI and concomitant AF, rising from 21.0% in 2000–2001 to 75.4% in 2020–2021, see *Figure 2*. The prescription of warfarin peaked at 37.9% in 2014–2015 and subsequently declined to 8.4% in 2020–2021, shown in green in *Figure 2*. In contrast, DOAC prescribing increased from 0.0% in 2010–2011 to 66.0% in 2020–2021, following the approval of the first DOAC for AF in Sweden in 2011 ( shown in red in *Figure 2*). With the increased use of OACs, the prescription of OAC in combination with DAPT (triple therapy) rose from 0.4% in 2000–2001, peaked at 24.7% in 2018–2019, and subsequently declined to 17.5% in 2020–2021. Conversely, the prescription of OAC in combination with SAPT (dual antithrombotic treatment) steadily increased from 5.4% in 2000–2001 to 44.6% in 2020–2021.

### One-year outcomes over a 22 years period

**Figure 3:**
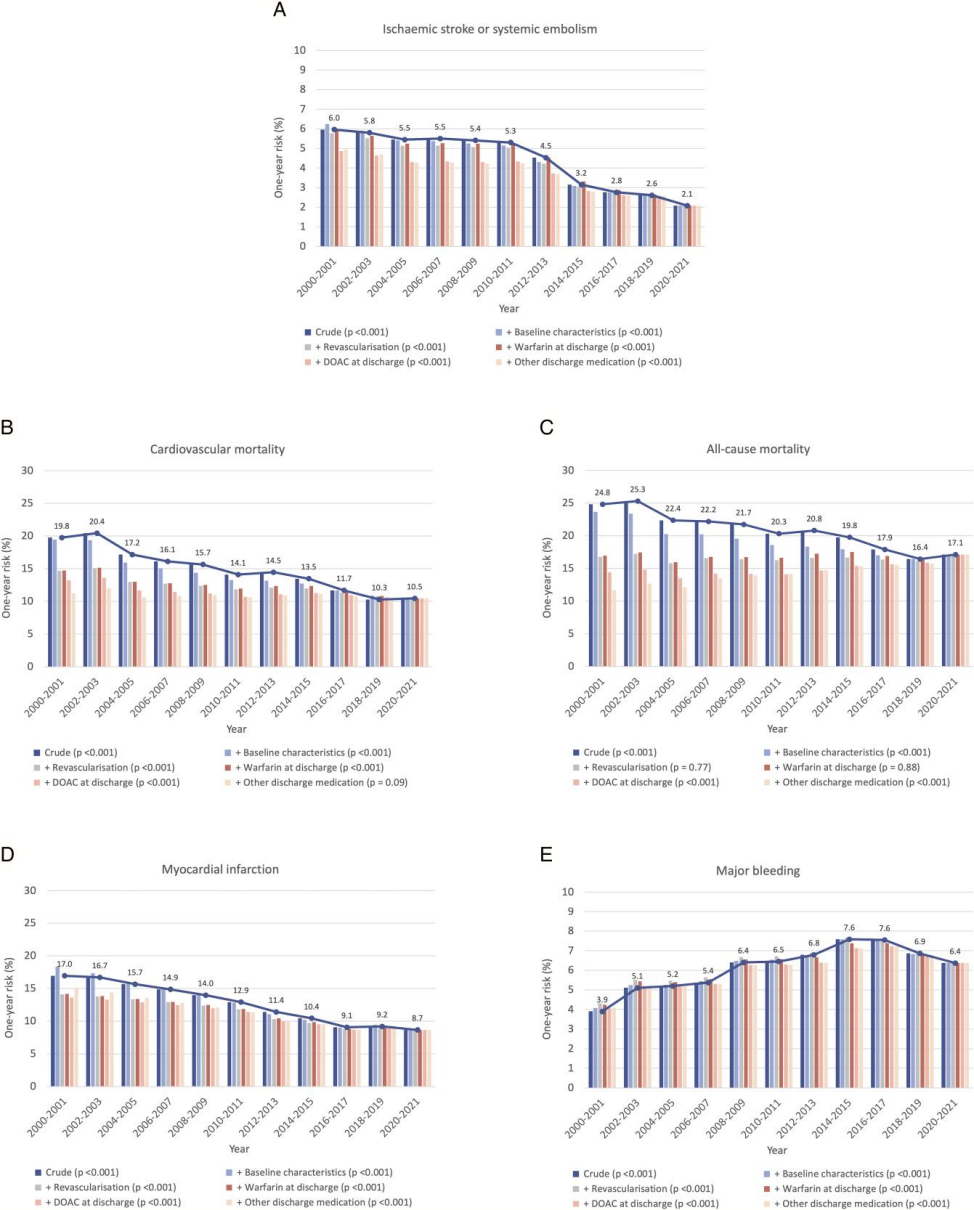
Stacked diagram of crude and standardized 1-year risk of (*A*) ischaemic stroke or systemic embolism, (*B*) cardiovascular mortality, (*C*) all-cause mortality, (*D*) recurrent MI, and (*E*) major bleeding in cases with MI and concomitant AF. *P*-value for trend over time is shown.

**Figure 4:**
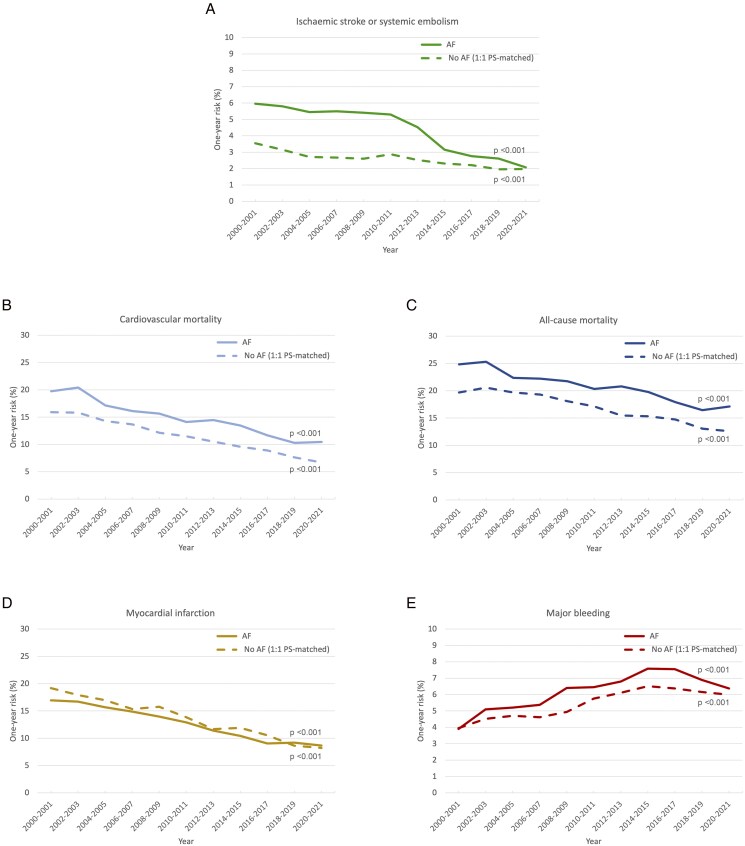
One-year crude risk curves of (*A*) ischaemic stroke or systemic embolism, (*B*) cardiovascular mortality, (*C*) all-cause mortality, (*D*) recurrent MI, and (*E*) major bleeding among cases with MI and concomitant AF, and in 1:1 propensity score-matched cases with MI but without AF between 2000 and 2021. P-value for trend over time is shown.

Among patients with acute MI and AF, the 1-year risk of ischaemic stroke or systemic embolism after hospital discharge decreased continuously from 6.0% in 2000–2001 to 2.1% in 2020–2021 (*Figure 3A*, Supplementary material online, *Figure S3*), reaching levels similar to those of the 1:1 propensity score-matched acute MI patients without AF (*Figure 4A dashed line*, Supplementary material online, *Table S5*). The 1-year risk of stroke or systemic embolism in patients with MI and AF in 2000–2001 was 6.2% when standardized for demographics and comorbidities, 5.8% after adding hospital revascularization and 5.9% after adding warfarin treatment. Adding standardization for DOAC treatment resulted in a 1-year risk of stroke or systemic embolism of 4.9%. Furthermore, including secondary preventive medication increased this number to 5.0%. The added standardizations are depicted as sequential bars in *Figure 3A*.

The risk of major bleeding during the first year after discharge increased from 3.9% in 2000–2001 to a peak of 7.6% in 2016–2017, before declining to 6.4% in 2020–2021 (*Figure 3E*). The observed increase in major bleeding was primarily driven by higher rates of gastrointestinal bleeding and other bleeding, although an increase in intracranial bleeding was also noted (see Supplementary material online, *Figure S4*). The 1-year risk of major bleeding in 2000–2001 was 4.3% when standardized for revascularization, 4.2% when adding warfarin and 4.1% when adding DOAC (*Figure 3E*).

There was a decrease in the 1-year risk of CV mortality (from 19.8% to 10.5%), all-cause mortality (from 24.8% to 17.1%), and MI (from 17.0% to 8.7%) during the study period among patients with acute MI and AF (*Figure 3B*, Supplementary material online, *Figure S3*). In the sensitivity analysis, CV mortality among patients with MI and AF decreased over the study period but never reached the levels observed in the 1:1 propensity score-matched patients without AF (*Figure 4B* solid and dashed lines, respectively). Model-based standardization suggested that reductions in CV mortality, all-cause mortality, and MI were largely associated with increased revascularization, with additional but more modest contributions related to OAC therapy (*Figure 3B-D*).

## Discussion

In this nationwide observational study of patients with acute MI and concurrent AF over two decades, we observed prescriptions of OACs and particularly DOACs at discharge increasing markedly following the approval of the first DOAC. In line with changes in recommendations of clinical practice guidelines over time, the use of triple therapy initially rose but later declined in favour of dual antithrombotic therapy with SAPT combined with a DOAC. Alongside evolving treatment practices, patient outcomes improved and 1-year risk of ischaemic stroke or systemic embolism decreased dramatically, reaching levels similar to those seen in propensity score-matched MI patients without AF. One-year risk of recurrent MI, CV mortality and all-cause mortality also decreased over the observation period. Among patients with acute MI and AF, the 1-year risk of major bleeding initially increased, reaching a peak that coincided with peak use of warfarin and triple therapy. At the latter part of the study period, we observed declining risks of major bleeding simultaneously with DOACs replacing warfarin and dual antithrombotic therapy replacing triple antithrombotic therapy.

AF in the coexistence of MI presents a therapeutic challenge in determining the optimal antithrombotic regimen and duration of treatment. For the first time, we were able to link evolving antithrombotic treatment strategies and practices to clinical outcomes in this context. Our findings show a temporal association between introduction of DOAC and lower risk of ischaemic stroke or systemic embolism and lower risk of CV and all-cause mortality, findings that were also observed in the standardized analyses. Furthermore, we observed that higher utilization of triple therapy and warfarin-based antithrombotic therapy coincided in time with higher rates of major bleeding, while the gradual adoption of DOAC-based dual antithrombotic therapy coincided with a decline in major bleeding events. Reassuringly, the risk of recurrent MI still declined in the recent years of the study, despite the observed shift from dual rather than triple antithrombotic therapy. Collectively, these findings provide real-world insights into the association between adoption of DOAC-based dual antithrombotic therapy and temporal changes in ischaemic and bleeding outcomes.

Despite the decrease in mortality among patients with acute MI and AF observed in this study, mortality in this patient group remained constantly higher than in the propensity-score matched MI population with no AF. It is well established that AF is associated with an increased mortality risk.^2,23^ This excess risk may partly reflect a higher burden of comorbidities, cardiovascular risk factors and frailty, as well as haemodynamic disturbances, arrhythmia-related complications, and structural cardiac changes associated with AF, which can adversely affect outcomes after MI and may not be fully captured in registry data. Recognizing this elevated risk in patients with both AF and MI is critical for optimizing follow-up and intensifying therapeutic strategies.

The pivotal RCTs in this field, including AUGUSTUS, RE-DUAL PCI, PIONEER AF-PCI, and ENTRUST-AF PCI, prompted new guidelines owing to the superior safety of DOAC-based dual antithrombotic therapy compared with triple antithrombotic therapy (mostly with warfarin).^15–18^ Although the populations in these trials differed from the present cohort in terms of patient selection and bleeding risk profiles, the reduction in major bleeding observed with the DOAC-based dual antithrombotic therapy in these trials is consistent with the findings of the present study. While these trials provided some indications of benefit with respect to bleeding outcomes, they were not designed to detect differences in ischaemic events.

To our knowledge, no previous study has evaluated the nationwide translational impact of clinical trial evidence and guideline updates on treatment patterns and outcomes in patients with acute MI and AF. Our findings demonstrate a clear shift in clinical practice that aligns temporally with the publication of trial evidence and subsequent guideline changes. This is encouraging, as previous research has shown that timely implementation of high-quality evidence is associated with reduced mortality.^24^ Importantly, despite 91% having a CHA_2_DS_2_-VASc score ≥3 points, we found approximately 25% of patients with MI and AF in the later part of the observation period did not receive DOAC treatment. Some of these patients may be ineligible to DOAC due to contraindications, adverse side effects, or personal preference. Still, it is possible that a significant proportion is not treated with DOAC due to persistent gaps in clinician awareness regarding the benefits of DOAC, or due to an overestimation of the risks associated with its use in combination with antiplatelet therapy.

A strength of this study is its nationwide coverage of hospitalized patients with acute MI and AF in Sweden over more than two decades, providing high external validity. However, the observational design limits causal inferences. Outcome were captured from the National Patient Register and the National Cause of Death Register using ICD-10 coding, which may be subject to misclassification, though these registers have previously been shown to be highly reliable.^21,22^ Although data completeness was high, residual confounding cannot be excluded. For example, the observed reduction in ischaemic stroke or systemic embolism cannot be fully attributed to OAC prescription, and part of the decline remains unexplained, suggesting the presence of other contributing variables not captured by the models. Such could be variability in frailty of the study population that goes beyond adjustment for baseline characteristics of age and comorbidities. Another limitation relates to the classification of diseases that may have undergone revisions during the study period. The definition of MI has been revised, and more sensitive diagnostic tools may have increased detection of milder MI cases. Similarly, the wider use of magnetic resonance imaging in diagnosing ischaemic stroke could have led to underreporting in earlier years. Further, discharge medication data reflects prescriptions at hospital discharge and may not reflect adherence or changes in antithrombotic therapy during the follow-up. Some patients may have switched or not adhered to prescribed treatment, although previous studies indicate OAC adherence to be generally high.^25^ Finally, patients who died during hospitalization were excluded, and outcomes were only captured after discharge, so early in-hospital thrombotic or bleeding events were not included, as information on in-hospital medication was unavailable.

## Conclusions

Among patients with acute MI and concomitant AF in Sweden between 2000 and 2021, there has been a clear shift towards treatment with DOACs in combination with single antiplatelet therapy. This shift was observed simultaneously with reduced risks of ischaemic stroke or systemic embolism, death and major bleeding. These findings provide real-world data supporting DOAC based dual therapy, for the large majority of patients with AF in the context of acute MI.

## Supplementary Material

pvag027_Supplementary_Data

## Data Availability

Data are available upon reasonable request. SWEDEHEART does not allow individual data sharing to third parties. Access to aggregated data might be granted following review by the SWEDEHEART steering committee. Such requests can be submitted to the SWEDEHEART steering committee for consideration. For contact details see: www.ucr.uu.se/swedeheart.
