# Supplementary material for: Implementation of novel antithrombotic treatment strategies and outcome improvements in patients with myocardial infarction and atrial fibrillation over 20 years: a nationwide cohort study
Source: Eur Heart J Cardiovasc Pharmacother. 2026 Apr 27;12(4):247–57. doi: 10.1093/ehjcvp/pvag027 (PMC13367244; doi:10.1093/ehjcvp/pvag027)
Supplement: pvag027_Supplementary_Data [file pvag027_supplementary_data.pdf]

Supplementary material

Implementation of novel antithrombotic treatment strategies and outcome improvements in patients with myocardial infarction and atrial fibrillation over 20 years: a nationwide cohort study

Contents

Supplementary Figure 1 2
Supplementary Table 1 4
Supplementary Table 2 5
Supplementary Table 3 6
Supplementary Table 4 7
Supplementary Figure 2 8
Supplementary Table 5 9
Supplementary Figure 3 11
Supplementary Figure 4 12
Supplementary Table 6 13

Supplementary Figure 1. STROBE statement

STROBE Statement – checklist of items that should be included in reports of observational studies

	Item No	Recommendation
**Title and abstract**	1	(*a*) Indicate the study's design with a commonly used term in the title or the abstract
		(*b*) Provide in the abstract an informative and balanced summary of what was done and what was found
**Introduction**		
Background/rationale	2	Explain the scientific background and rationale for the investigation being reported
Objectives	3	State specific objectives, including any prespecified hypotheses
**Methods**		
Study design	4	Present key elements of study design early in the paper
Setting	5	Describe the setting, locations, and relevant dates, including periods of recruitment, exposure, follow-up, and data collection
Participants	6	(*a*) *Cohort study*—Give the eligibility criteria, and the sources and methods of selection of participants. Describe methods of follow-up<br>*Case-control study*—Give the eligibility criteria, and the sources and methods of case ascertainment and control selection. Give the rationale for the choice of cases and controls<br>*Cross-sectional study*—Give the eligibility criteria, and the sources and methods of selection of participants
		(*b*) *Cohort study*—For matched studies, give matching criteria and number of exposed and unexposed<br>*Case-control study*—For matched studies, give matching criteria and the number of controls per case
Variables	7	Clearly define all outcomes, exposures, predictors, potential confounders, and effect modifiers. Give diagnostic criteria, if applicable
Data sources/ measurement	8*	For each variable of interest, give sources of data and details of methods of assessment (measurement). Describe comparability of assessment methods if there is more than one group
Bias	9	Describe any efforts to address potential sources of bias
Study size	10	Explain how the study size was arrived at
Quantitative variables	11	Explain how quantitative variables were handled in the analyses. If applicable, describe which groupings were chosen and why
Statistical methods	12	(*a*) Describe all statistical methods, including those used to control for confounding
		(*b*) Describe any methods used to examine subgroups and interactions
		(*c*) Explain how missing data were addressed
		(*d*) *Cohort study*—If applicable, explain how loss to follow-up was addressed<br>*Case-control study*—If applicable, explain how matching of cases and controls was addressed<br>*Cross-sectional study*—If applicable, describe analytical methods taking account of sampling strategy
		(*e*) Describe any sensitivity analyses

Supplementary Figure 1 (continued). STROBE statement

✓ **Results**		
Participants	13*	(a) Report numbers of individuals at each stage of study—eg numbers potentially eligible, examined for eligibility, confirmed eligible, included in the study, completing follow-up, and analysed
		(b) Give reasons for non-participation at each stage
		(c) Consider use of a flow diagram
Descriptive data	14*	(a) Give characteristics of study participants (eg demographic, clinical, social) and information on exposures and potential confounders
		(b) Indicate number of participants with missing data for each variable of interest
		(c) *Cohort study*—Summarise follow-up time (eg, average and total amount)
Outcome data	15*	*Cohort study*—Report numbers of outcome events or summary measures over time
		Case-control study—Report numbers in each exposure category, or summary measures of exposure
		Cross-sectional study—Report numbers of outcome events or summary measures
Main results	16	(*a*) Give unadjusted estimates and, if applicable, confounder-adjusted estimates and their precision (eg, 95% confidence interval). Make clear which confounders were adjusted for and why they were included
		(*b*) Report category boundaries when continuous variables were categorized
		(*c*) If relevant, consider translating estimates of relative risk into absolute risk for a meaningful time period
Other analyses	17	Report other analyses done—eg analyses of subgroups and interactions, and sensitivity analyses
✓ **Discussion**		
Key results	18	Summarise key results with reference to study objectives
Limitations	19	Discuss limitations of the study, taking into account sources of potential bias or imprecision. Discuss both direction and magnitude of any potential bias
Interpretation	20	Give a cautious overall interpretation of results considering objectives, limitations, multiplicity of analyses, results from similar studies, and other relevant evidence
Generalisability	21	Discuss the generalisability (external validity) of the study results
✓ **Other information**		
Funding	22	Give the source of funding and the role of the funders for the present study and, if applicable, for the original study on which the present article is based

*Give information separately for cases and controls in case-control studies and, if applicable, for exposed and unexposed groups in cohort and cross-sectional studies.

Note: An Explanation and Elaboration article discusses each checklist item and gives methodological background and published examples of transparent reporting. The STROBE checklist is best used in conjunction with this article (freely available on the Web sites of PLoS Medicine at http://www.plosmedicine.org/, Annals of Internal Medicine at http://www.annals.org/, and Epidemiology at http://www.epidem.com/). Information on the STROBE Initiative is available at www.strobe-statement.org.

Supplementary Table 1. Data sources and the International Code of Disease, tenth revision (ICD-10) applied to identify comorbidities and comedication.

Variable	Data source	ICD-10
Calendar year	SWEDEHEART	-
Age	SWEDEHEART	-
Sex	SWEDEHEART	-
Obesity	SWEDEHEART	-
Smoking	SWEDEHEART	-
Atrial fibrillation	SWEDEHEART, NPR	I48
Hypertension	SWEDEHEART, NPR	I10, I11, I12, I13, I15
Diabetes	SWEDEHEART, NPR	E10, E11, E12, E13, E14
Heart failure	SWEDEHEART, NPR	I42, I50, I110, I255, I130, I132, K761
COPD	NPR	J43, J44
Prior MI	SWEDEHEART, NPR	I21, I22, I252
Prior ischemic stroke	SWEDEHEART, NPR	I63
Prior systemic embolism	NPR	I74
Peripheral vascular disease	NPR	I70, I71, I72, I73
Cancer (within 3 years)	NPR	C
Prior major bleeding	NPR	I60, I61, I62, S064, S065, S066, K226, K250, K252, K254, K256, K260, K262, K264, K266, K270, K272, K274, K276, K280, K282, K284, K286, K290, K625, K661, K920, K921, K922, I850, I983, N02, R319, N939, N950, N501A, H113, H313, H356, H431, H450, H922, I312, J942, M250, R04, R58, T810, D500, D629, T792
Type of MI (NSTEMI, STEMI)	SWEDEHEART	-
New-onset atrial fibrillation	SWEDEHEART	-
Thrombolysis	SWEDEHEART	-
Coronary angiography	SWEDEHEART	-
PCI	SWEDEHEART	-
Acetylsalicylic acid	SWEDEHEART	-
$P2Y_{12}$ inhibitor	SWEDEHEART	-
Warfarin	SWEDEHEART	-
DOAC	SWEDEHEART	-
Beta blockers	SWEDEHEART	-
ACE inhibitors/ARB	SWEDEHEART	-
Statins	SWEDEHEART	-
Ezetimibe	SWEDEHEART	-

Abbreviations: ACE, angiotensin-converting enzyme; AF, atrial fibrillation; ARB, angiotensin receptor blockers; BMI, body mass index; CABG, coronary artery bypass graft surgery; COPD, chronic obstructive pulmonary disease; DOAC, direct oral anticoagulants; MI, myocardial infarction; NPR, National Patient Register; NSTEMI, non-ST-segment elevation myocardial infarction; PCI, percutaneous coronary intervention; STEMI, ST-segment elevation myocardial infarction.

Supplementary Table 2. Data sources and the International Code of Disease, tenth revision (ICD-10) applied to identify outcomes.

Variable	Data source	ICD-10 (primary or secondary diagnosis)
Ischaemic stroke	NPR	I63
Systemic embolism	NPR	I74
All-cause mortality	National Cause of Death Registry	-
Cardiovascular mortality	National Cause of Death Registry	I00 – I99
Myocardial infarction	SWEDEHEART, NPR	I21, I22<br>(Myocardial infarction within 30 days after discharge was defined as a new myocardial infarction recorded in SWEDEHEART if occurring more than 2 days after discharge. Myocardial infarction after 30 days was defined as readmission of a new myocardial infarction in the NPR).
Major bleeding	NPR	I60, I61, I62, S064, S065, S066, K226, K250, K252, K254, K256, K260, K262, K264, K266, K270, K272, K274, K276, K280, K282, K284, K286, K290, K625, K661, K920, K921, K922, I850, I983, N02, R319, N939, N950, N501A, H113, H313, H356, H431, H450, H922, I312, J942, M250, R04, R58, T810, D500, D629, T792

Abbreviations: NPR, national patient register.

Supplementary Table 3. Standardisation steps and included variables .

Standardisation steps	Variables included
1. Demographics and comorbidities	Age, sex, hypertension, diabetes, heart failure, COPD, prior MI, prior stroke, peripheral vascular disease, cancer (within 3 years), prior major bleeding, type of MI (STEMI, NSTEMI), new-onset AF, eGFR
2. Revascularization	Step 1 + thrombolysis, coronary angiography, PCI, CABG
3. Warfarin	Step 2 + warfarin at discharge
4. DOAC	Step 3 + DOAC at discharge
5. Secondary preventive medication	Step 4 + aspirin, $P2Y_{12}$ inhibitors, ACE inhibitors, ARB

Supplementary Table 4. Missing data .

Variable	Missing
Age	0 (0.0%)
Sex	0 (0.0%)
Hypertension	0 (0.0%)
Diabetes	0 (0.0%)
Heart failure	0 (0.0%)
COPD	0 (0.0%)
Prior MI	0 (0.0%)
Prior stroke	0 (0.0%)
Peripheral vascular disease	0 (0.0%)
Cancer (within 3 years)	0 (0.0%)
Prior major bleeding	0 (0.0%)
Type of MI	2,824 (0.8%)
New-onset AF	3,419 (1.0%)
eGFR	58,047 (16.5%)
Thrombolysis	1,863 (0.5%)
Coronary angiography	0 (0.0%)
PCI	0 (0.0%)
CABG	0 (0.0%)
Warfarin	2,540 (0.7%)
Aspirin, $P2Y_{12}$ inhibitors	1,855 (0.5%)
ACE inhibitors, ARB	0 (0.0%)
Beta blockers	1,866 (0.5%)
Statins	2,371 (0.7%)
Ezetimibe	3,479 (1.0%)

Supplementary Figure 2. Covariate balance before and after 1:1 propensity score matching between AF and non-AF patient cohort with acute MI.

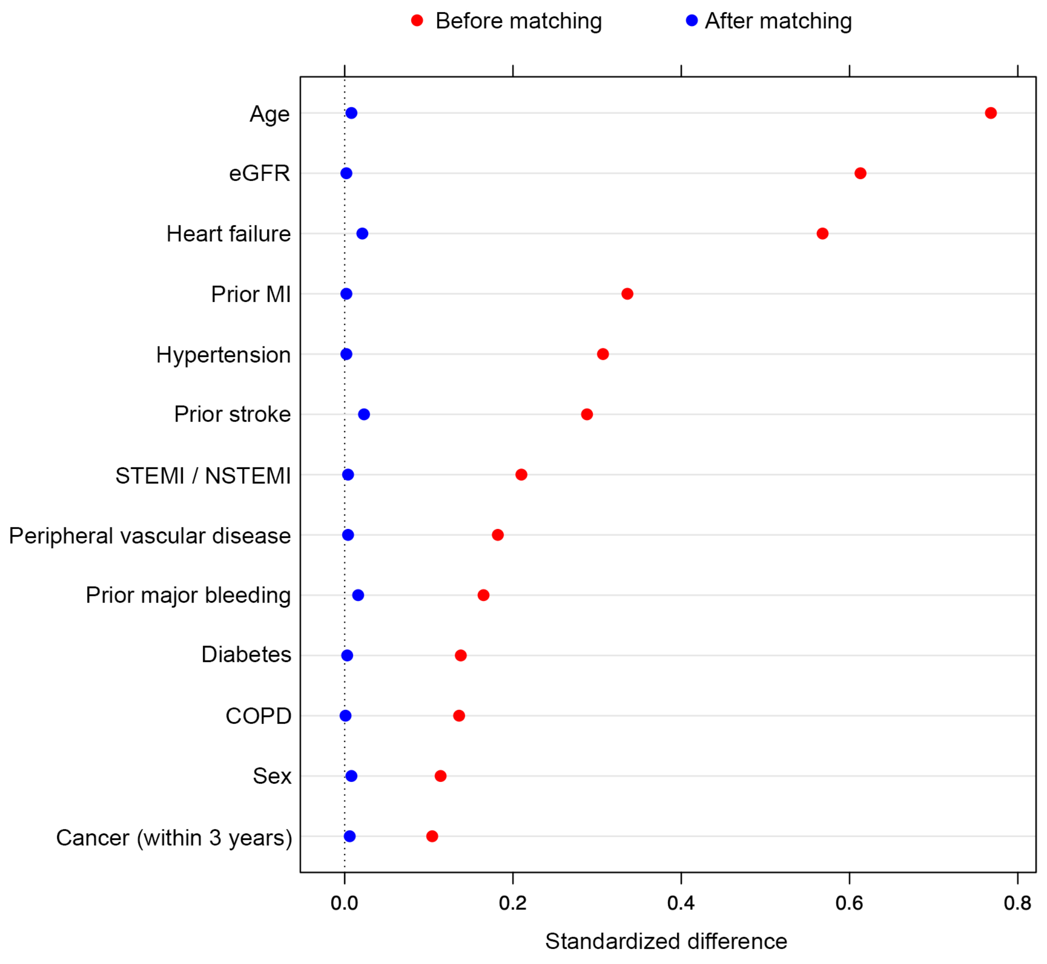

Supplementary Table 5. Baseline demographics and characteristics of patients with MI and AF, and of 1:1 propensity score-matched patients without AF.

Characteristics	Atrial fibrillation (n = 71,513)	1:1 PS-matched patients with no atrial fibrillation (n = 71,513)
Demographics		
Age, years, median (IQR)	79 (72-84)	79 (72-84)
Sex, male, n (%)	43,516 (60.9%)	43,231 (60.5%)
Obesity (BMI ≥30 kg/m^2), n (%) [n = 99,961]	10,460 (21.0%)	9,385 (18.7%)
Smoking, n (%) [n = 128,354]	8,179 (12.8%)	8,965 (13.9%)
Medical history, n (%)		
Hypertension	49,415 (69.1%)	49,498 (69.2%)
Diabetes	21,446 (30.0%)	21,554 (30.1%)
Heart failure	29,152 (40.8%)	28,515 (39.9%)
COPD	7,295 (10.2%)	7,266 (10.2%)
Prior MI	28,219 (39.5%)	28,289 (39.6%)
Prior stroke	13,850 (19.4%)	13,290 (18.6%)
Prior systemic embolism	874 (1.2%)	496 (0.7%)
Cancer (within 3 years)	4,200 (5.9%)	4,108 (5.7%)
Peripheral vascular disease	6,928 (9.7%)	6,855 (9.6%)
Prior major bleeding	6,640 (9.3%)	6,339 (8.9%)
CHA_2DS_2-VASc score, n (%)		
1 point	1,683 (2.4%)	1,807 (2.5%)
2 points	4,580 (6.4%)	4,584 (6.4%)
3 points	9,704 (13.6%)	9,859 (13.8%)
≥4 points	55,546 (77.7%)	55,263 (77.3%)
In-hospital course, n (%)		
NSTEMI [n = 141,750]	53,020 (74.8%)	53,188 (75.0%)
STEMI [n = 141,750]	17,833 (25.1%)	17,709 (25.0%)
New-onset AF	35,859 (50.1%)	n/a
Thrombolysis	2,766 (3,9%)	3,090 (4.3%)
Coronary angiography	37,185 (52.0%)	39,577 (55.3%)
PCI	27,308 (38.2%)	30,419 (42.5%)

CABG	1,987 (2.8%)	2,107 (2.9%)
Medication at discharge, n (%)		
Warfarin [n = 142,454]	16,517 (23.3%)	2,296 (3.2%)
DOAC	9,973 (13.9%)	502 (0.7%)
Acetylsalicylic acid [n = 142,214]	52,139 (73.3%)	64,905 (91.3%)
$P2Y_{12}$ inhibitors [n = 142,199]	39,002 (54.9%)	46,683 (65.7%)
Beta blockers [n = 142,179]	61,345 (86.3%)	60,245 (84.7%)
ACE inhibitor/ARB	49,567 (69.3%)	49,798 (69.6%)
Statins [n = 141,923]	48,936 (69.0%)	52,482 (74.0%)
Ezetimibe [n = 141,627]	1,517 (2.1%)	1,576 (2.2%)

Values are in median (interquartile range [IQR]) for continuous variables and numbers (%) for categorical variables. Numbers within square brackets indicate number patients with available data and no missing values. Abbreviations: ACE, angiotensin-converting enzyme; AF, atrial fibrillation; ARB, angiotensin receptor blockers; BMI, body mass index; CHA_2DS_2-VASc, congestive heart failure, hypertension, age, diabetes, stroke, vascular disease, sex; CABG, coronary artery bypass graft surgery; COPD, chronic obstructive pulmonary disease; DOAC, direct oral anticoagulants; MI, myocardial infarction; NSTEMI, non-ST-segment elevation myocardial infarction; PCI, percutaneous coronary intervention; PS, propensity score; STEMI, ST-segment elevation myocardial infarction

Supplementary Figure 3. Kaplan-Meier crude risk curves for (A) ischaemic stroke or systemic embolism, (B) cardiovascular mortality, (C) all-cause mortality, (D) recurrent MI, and (E) major bleeding in cases with acute MI and concomitant AF between 2000 – 2021.

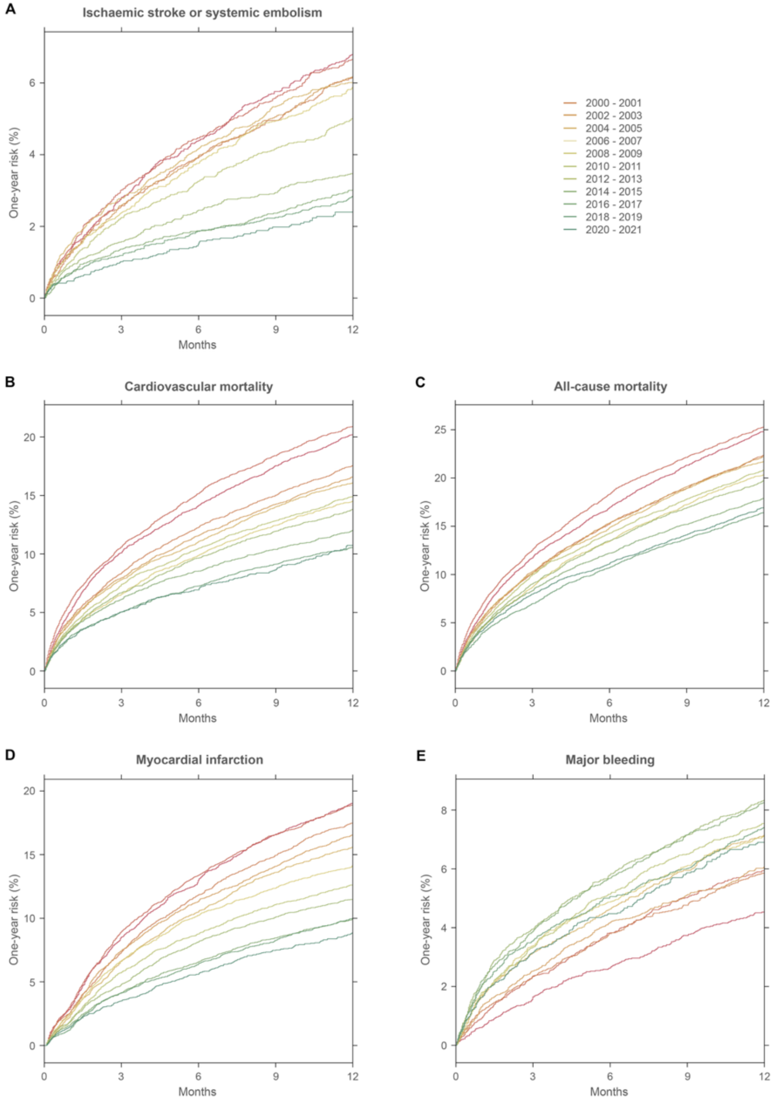

Supplementary Figure 4. One-year crude risk of (A) intracranial bleeding, (B) gastrointestinal bleeding, and (C) other bleeding events in patients with MI and AF, and in 1:1 propensity score-matched patients with MI but without AF. P-value for trend over time.

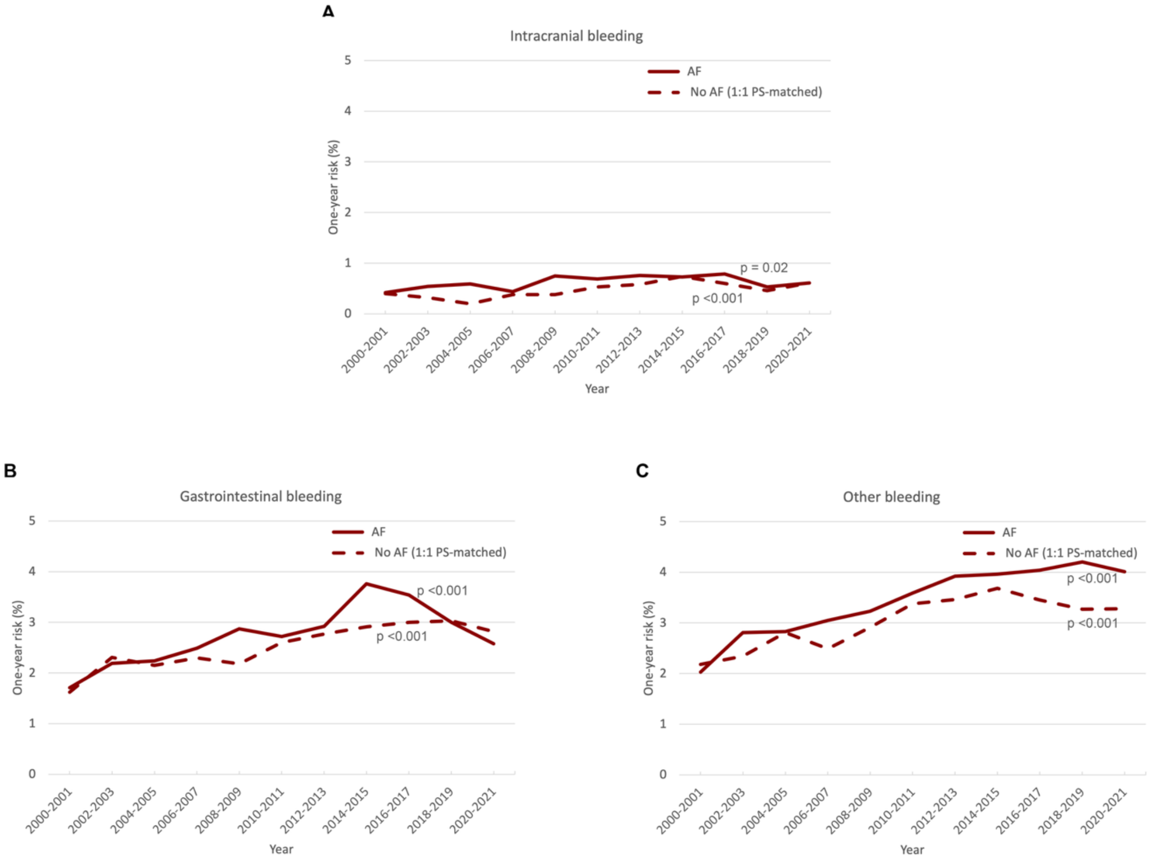

Supplementary Table 6. Detailed information about antithrombotic agents in 2-year blocks for patients with acute MI and concomitant AF.

Characteristics	2000 – 2001	2002 – 2003	2004 – 2005	2006 – 2007	2008 – 2009	2010 – 2011	2012 – 2013	2014 – 2015	2016 – 2017	2018 – 2019	2020 – 2021
	(n = 5,970)	(n = 6,789)	(n = 6,789)	(n = 7,107)	(n = 6,909)	(n = 6,679)	(n = 6,688)	(n = 6,408)	(n = 6,334)	(n = 6,169)	(n = 5,671)
**Only SAPT**	3,779	3,482	2,720	2,169	1,651	1,382	1,104	759	597	454	382
Aspirin	3,646 (96.5%)	3,241 (93.1%)	2,514 (92.4%)	1,984 (91.5%)	1,496 (90.6%)	1,241 (89.8%)	922 (83.5%)	613 (80.8%)	483 (80.9%)	368 (81.1%)	306 (80.1%)
Clopidogrel	133 (3.5%)	223 (6.4%)	190 (7.0%)	179 (8.3%)	147 (8.9%)	130 (9.4%)	140 (12.7%)	105 (13.8%)	85 (14.2%)	60 (13.2%)	64 (16.8%)
Ticagrelor/prasugrel	0 (0.0%)	0 (0.0%)	0 (0.0%)	0 (0.0%)	0 (0.0%)	3 (0.2%)	33 (3.0%)	37 (4.9%)	25 (4.2%)	24 (5.3%)	11 (2.9%)
Other/unknown $P2Y_{12}$ type	0 (0.0%)	18 (0.5%)	16 (0.6%)	6 (0.3%)	8 (0.5%)	8 (0.6%)	9 (0.8%)	4 (0.5%)	4 (0.7%)	2 (0.4%)	1 (0.3%)
**Only DAPT**	319	1240	2312	3138	3360	3315	3051	2286	1668	1359	912
Aspirin	319 (100.0%)	1240 (100.0%)	2312 (100.0%)	3138 (100.0%)	3360 (100.0%)	3315 (100.0%)	3051 (100.0%)	2286 (100.0%)	1668 (100.0%)	1359 (100.0%)	912 (100.0%)
Clopidogrel	319 (100.0%)	1240 (100.0%)	2266 (98.0%)	3091 (98.5%)	3333 (99.2%)	3201 (96.6%)	1888 (61.9%)	963 (42.1%)	526 (31.5%)	426 (31.3%)	274 (30.0%)
Ticagrelor/prasugrel	0 (0.0%)	0 (0.0%)	0 (0.0%)	0 (0.0%)	1 (0.0%)	87 (2.6%)	1137 (37.3%)	1306 (57.1%)	1131 (67.8%)	922 (67.8%)	630 (69.1%)
Other/unknown $P2Y_{12}$ type	0 (0.0%)	0 (0.0%)	46 (2.0%)	47 (1.5%)	26 (0.8%)	27 (0.8%)	26 (0.9%)	17 (0.7%)	11 (0.7%)	11 (0.8%)	8 (0.9%)
**Warfarin + SAPT**	323	415	481	632	674	656	987	1,009	671	353	258
Aspirin	293 (90.7%)	345 (83.1%)	344 (71.5%)	416 (65.8%)	441 (65.4%)	468 (71.3%)	524 (53.1%)	348 (34.5%)	268 (39.9%)	113 (32.0%)	66 (25.6%)
Clopidogrel	30 (9.3%)	62 (14.9%)	131 (27.2%)	216 (34.2%)	230 (34.1%)	185 (28.2%)	375 (38.0%)	509 (50.4%)	275 (41.0%)	172 (48.7%)	161 (62.4%)
Ticagrelor/prasugrel	0 (0.0%)	0 (0.0%)	0 (0.0%)	0 (0.0%)	0 (0.0%)	2 (0.3%)	85 (8.6%)	151 (15.0%)	126 (18.8%)	68 (19.3%)	31 (12.0%)
Other/unknown $P2Y_{12}$ type	0 (0.0%)	8 (1.9%)	6 (1.2%)	0 (0.0%)	3 (0.4%)	1 (0.2%)	3 (0.3%)	1 (0.1%)	2 (0.3%)	0 (0.0%)	0 (0.0%)
**Warfarin + DAPT**	26	89	123	206	362	620	816	924	766	297	98
Aspirin	26 (100%)	89 (100%)	123 (100%)	206 (100%)	362 (100%)	620 (100%)	816 (100%)	924 (100%)	766 (100%)	297 (100%)	98 (100%)
Clopidogrel	26 (100%)	89 (100%)	122 (99.2%)	204 (99.0%)	362 (100%)	614 (99.0%)	707 (86.6%)	856 (92.6%)	742 (96.9%)	279 (93.9%)	91 (92.9%)
Ticagrelor/prasugrel	0 (0.0%)	0 (0.0%)	0 (0.0%)	0 (0.0%)	0 (0.0%)	5 (0.8%)	107 (13.1%)	64 (6.9%)	23 (3.0%)	17 (5.7%)	7 (7.1%)
Other/unknown $P2Y_{12}$ type	0 (0.0%)	0 (0.0%)	1 (0.8%)	2 (1.0%)	0 (0.0%)	1 (0.2%)	2 (0.2%)	4 (0.4%)	1 (0.1%)	1 (0.3%)	0 (0.0%)
**Only DOAC**	0	0	0	0	0	0	10	225	348	483	570
Dabigatran	-	-	-	-	-	-	10 (100%)	20 (8.9%)	32 (8.3%)	27 (5,6%)	26 (4.6%)
Rivaroxaban	-	-	-	-	-	-	0 (0.0%)	61 (27.1%)	75 (19.5%)	75 (15.5%)	65 (11.4%)
Apixaban	-	-	-	-	-	-	0 (0.0%)	144 (64.0%)	277 (72.1%)	379 (78.5%)	457 (80.2%)
Edoxaban	-	-	-	-	-	-	0 (0.0%)	0 (0.0%)	0 (0.0%)	2 (0.4%)	22 (3.9%)
**DOAC + SAPT**	0	0	0	0	0	0	12	316	988	1619	2275

Dabigatran	-	-	-	-	-	-	12 (100%)	48 (15.2%)	110 (11.1%)	155 (9.6%)	135 (5.9%)
Rivaroxaban	-	-	-	-	-	-	0 (0.0%)	71 (22.5%)	183 (18.5%)	327 (20.2%)	259 (11.4%)
Apixaban	-	-	-	-	-	-	0 (0.0%)	197 (62.3%)	695 (70.3%)	1116 (68.9%)	1817 (79.9%)
Edoxaban	-	-	-	-	-	-	0 (0.0%)	0 (0.0%)	0 (0.0%)	21 (1.3%)	64 (2.8%)
Aspirin	-	-	-	-	-	-	6 (50%)	128 (40.5%)	287 (29.0%)	373 (23.0%)	368 (12.2%)
Clopidogrel	-	-	-	-	-	-	4 (33.3%)	124 (39.2%)	444 (44.9%)	842 (52.0%)	1543 (67.8%)
Ticagrelor/prasugrel	-	-	-	-	-	-	2 (16.7%)	64 (20.3%)	257 (26.0%)	404 (25.0%)	362 (15.9%)
Other/unknown $P2Y_{12}$ type	-	-	-	-	-	-	0 (0.0%)	0 (0.0%)	0 (0.0%)	0 (0.0%)	2 (0.1%)
**DOAC + DAPT**	0	0	0	0	0	1	16	171	773	1228	898
Dabigatran	-	-	-	-	-	1 (100%)	16 (100%)	16 (9.4%)	84 (10.9%)	115 (9.4%)	52 (5.8%)
Rivaroxaban	-	-	-	-	-	0 (0.0%)	0 (0.0%)	56 (32.7%)	155 (20.1%)	182 (14.8%)	88 (9.8%)
Apixaban	-	-	-	-	-	0 (0.0%)	0 (0.0%)	99 (57.9%)	534 (69.1%)	921 (75.0%)	747 (83.2%)
Edoxaban	-	-	-	-	-	0 (0.0%)	0 (0.0%)	0 (0.0%)	0 (0.0%)	10 (0.8%)	11 (1.2%)
Aspirin	-	-	-	-	-	1 (100%)	16 (100%)	171 (100%)	773 (100%)	1228 (100%)	898 (100%)
Clopidogrel	-	-	-	-	-	1 (100%)	12 (75%)	154 (90.1%)	706 (91.3%)	1150 (93.6%)	851 (94.8%)
Ticagrelor/prasugrel	-	-	-	-	-	0 (0.0%)	4 (25.0%)	16 (9.4%)	67 (8.7%)	77 (6.3%)	46 (5.1%)
Other/unknown $P2Y_{12}$ type	-	-	-	-	-	0 (0.0%)	0 (0.0%)	1 (0.6%)	0 (0.0%)	1 (0.1%)	1 (0.1%)

Values are in median (interquartile range [IQR]) for continuous variables and numbers (%) for categorical variables. Information of missing data is available in Supplementary Table 5. Abbreviations: ACE, angiotensin-converting enzyme; AF, atrial fibrillation; ARB, angiotensin receptor blockers; BMI, body mass index; CHA2DS2-VASc, congestive heart failure, hypertension, age, diabetes, stroke, vascular disease, sex; CABG, coronary artery bypass graft surgery; COPD, chronic obstructive pulmonary disease; DOAC, direct oral anticoagulants; MI, myocardial infarction; NSTEMI, non-ST-segment elevation myocardial infarction; PCI, percutaneous coronary intervention; STEMI, ST-segment elevation myocardial infarct
